# Disruption of *ClOSD1* leads to both somatic and gametic ploidy doubling in watermelon

**DOI:** 10.1093/hr/uhae288

**Published:** 2024-10-15

**Authors:** Wenyu Pang, Wenbing He, Jing Liang, Qiaran Wang, Shengcan Hou, Xiaodan Luo, Junhua Li, Jiafa Wang, Shujuan Tian, Li Yuan

**Affiliations:** State Key Laboratory of Crop Stress Resistance and High-Efficiency Production, College of Horticulture, Northwest A&F University, 3 Taicheng Road, Yangling 712100, Shaanxi, China; State Key Laboratory of Crop Stress Resistance and High-Efficiency Production, College of Horticulture, Northwest A&F University, 3 Taicheng Road, Yangling 712100, Shaanxi, China; State Key Laboratory of Crop Stress Resistance and High-Efficiency Production, College of Horticulture, Northwest A&F University, 3 Taicheng Road, Yangling 712100, Shaanxi, China; State Key Laboratory of Crop Stress Resistance and High-Efficiency Production, College of Horticulture, Northwest A&F University, 3 Taicheng Road, Yangling 712100, Shaanxi, China; Melon Institute, Kaifeng Academy of Agriculture and Forestry Sciences, Xinghuaying Street, 475000, Kaifeng, China; Melon Institute, Kaifeng Academy of Agriculture and Forestry Sciences, Xinghuaying Street, 475000, Kaifeng, China; Melon Institute, Kaifeng Academy of Agriculture and Forestry Sciences, Xinghuaying Street, 475000, Kaifeng, China; State Key Laboratory of Crop Stress Resistance and High-Efficiency Production, College of Horticulture, Northwest A&F University, 3 Taicheng Road, Yangling 712100, Shaanxi, China; State Key Laboratory of Crop Stress Resistance and High-Efficiency Production, College of Horticulture, Northwest A&F University, 3 Taicheng Road, Yangling 712100, Shaanxi, China; State Key Laboratory of Crop Stress Resistance and High-Efficiency Production, College of Horticulture, Northwest A&F University, 3 Taicheng Road, Yangling 712100, Shaanxi, China

Dear Editor,

Apomixis is a reproductive mode with significant potential in fixed heterosis, which relies on mitosis instead of meiosis (*MiMe*) to produce diploid gametes identical to the genetic composition of the maternal parent [1]. *MiMe*, initially established in *Arabidopsis thaliana* and subsequently applied successfully to rice [2, 3], hinges crucially on the formation of unreduced gametes. This milestone was first reached through the identification of the *Arabidopsis OMISSION OF SECOND DIVISION1* (*OSD1*) gene, known for its role as a negative regulator of the anaphase promoting complex (APC/C) [4]. Deletion of *OSD1* leads to premature termination of meiosis post the first division, likely due to hyperactivation of APC/C. Consequently, *Atosd1* mutant plants exhibit a complete absence of the meiotic second division, resulting in significantly elevated production rates (100% in males and 85% in females) of diploid spores and polyploid offspring [4, 5]. Additionally, the *AtOSD1* homolog *UV-B INSENSITIVE4* (*UVI4*) primarily functions at the G2-M phase, contributing to somatic ploidy regulation by negatively regulating APC/C activity [6]. Certain species, such as Cucurbitaceae, tomato, and potato, possess only a single homologous gene for *OSD1* and *UVI4* (Fig. 1A), raising questions about the functional divergence of *OSD1* in these crops compared to model plants. Thus, we conducted a related study in the Cucurbitaceae crop, watermelon (*Citrullus lanatus*).

**Figure 1:**
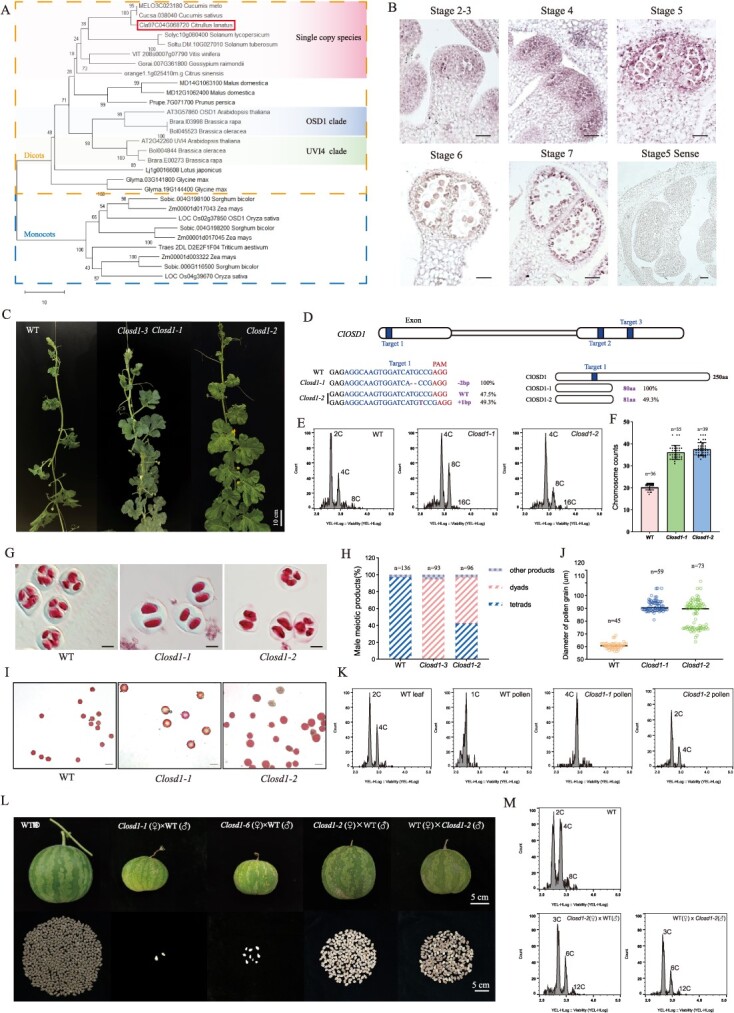
Disruption of *ClOSD1* leads to both somatic and gametic ploidy doubling in watermelon. (A) Phylogenetic tree depicting OSD1 predicted proteins from 19 monocot and dicot plant species. (B) *In situ* hybridization analysis of *ClOSD1* at different stages of watermelon male flower development. Scale bars: 50 μm. (C) Comparison of WT, *Closd1-1* and *Closd1-2* mutant plant phenotypes. (D) Schematic representation of *ClOSD1* gene structure, target sequence, and editing results in different *Closd1* mutants. (E) Flow cytometric analysis of *Closd1-1* and *Closd1-2* plants ploidy. (F) Chromosome count statistics in wild-type, *Closd1-1* and *Closd1-2* plants via immunofluorescence assay. (G) Male meiotic product and activity analysis in *Closd1-1*, *Closd1-2*, and WT plants. Scale bar: 20 μm. (H) Proportions of different male meiotic products in WT, *Closd1-1*, and *Closd1-2* mutant. (I) Pollen viability analysis using Alexander staining in mature pollen of WT, *Closd1-1*, and *Closd1-2* plants. Scale bar: 100 μm. (J) Statistics of mature pollen diameter in WT, *Closd1-1* and *Closd1-2*. (K) Ploidy determination of WT, *Closd1-1*, *and Closd1-2* mutant leaves and pollen. (L) Fruits and seeds of each hybrid combination of the different *Closd1* mutants. (M) Flow cytometry analysis of the progeny from reciprocal crosses between *Closd1-2* mutant.

Initially, through sequence alignment analysis employing AtOSD1 as the bait, we identified a singular candidate protein in watermelon, encoded by the *Cla97C04G068720* gene and designated as *ClOSD1* (Fig. 1A). It is noteworthy that another homolog of *AtOSD1*, *AtUVI4*, exists in the *Arabidopsis* genome (Fig. 1A). Both *AtUVI4* and *AtOSD1* function as inhibitors of the APC/C complex and collaboratively engage in cell cycle regulation. However, *AtUVI4* primarily governs mitosis and intracellular DNA replication. In *Atuvi4* mutants, somatic DNA replication proceeds and cellular DNA content escalates, while meiosis remains unaffected [6]. Interestingly, cross-species sequence alignment analysis unveiled that beyond Cucurbitaceae species, this gene also manifests as a singular copy in Cucumis, citrus, tomato, and potato (Fig. 1A). Certain species like soybean harbor two orthologous genes, yet these duplications emerged relatively later in evolutionary history. Most monocotyledonous plants possess two orthologous genes for *OSD1* expression, underscoring its species-specific nature throughout evolution.

Subcellular localization analysis revealed that ClOSD1 is located in the nucleus (Fig. S1). Expression profiling via RT-qPCR indicated the presence of *ClOSD1* transcripts in various vegetative and reproductive organs of watermelon. Notably, *ClOSD1* showed relatively diminished expression levels in roots and stems, while its expression surged in leaves and young fruits exhibiting active cell division (Fig. S1). These observations suggest a role for *ClOSD1* akin to *AtUVI4*, potentially involved in the regulation of the mitotic cell cycle [6]. During meiosis, *ClOSD1* displayed heightened expression during prophase I, followed by a gradual decline thereafter. Its expression remained relatively subdued during the transition from meiosis I to meiosis II, further decreasing during anaphase II (Fig. S1). This expression profile closely mirrors the nuanced regulation of unreduced gamete production by *Atosd1* during meiosis. *In situ* hybridization analysis of *ClOSD1* in male meiosis corroborated the RT-qPCR findings, with robust expression signals observed during meiosis I, followed by a subsequent decline (Fig. 1B). Collectively, these RT-qPCR and *in situ* hybridization results suggest that *ClOSD1* not only participates in germ cell meiosis but also modulates somatic cell mitosis.

To further elucidate the function of *ClOSD1*, we employed CRISPR/Cas9 technology to generate the mutant of *ClOSD1*. A total of eight T0-generation edited plants were obtained (Fig. S2). We examined potential off-target sites of the three sgRNAs in the gene-edited plants, and off-target mutations were excluded (Table S1, Table S3). *Closd1-2* and *Closd1-3* are heterozygous mutants, while *Closd1-1*, *Closd1-6*, *Closd1-7*, and *Closd1-8* are homozygous mutants. Additionally, *Closd1-4* and *Closd1-5* are biallelic mutants (Fig. S2). All these mutations result in varying degrees of protein alterations, with homozygous and biallelic mutants exhibiting identical phenotypic characteristics. Therefore, we selected *Closd1-1* and the heterozygous mutant line *Closd1–2* as representatives for phenotypic display and analysis (Fig. 1D).

Unexpectedly, a striking enhancement in field growth was observed in the *Closd1-1* and *Closd1-2* plants compared to the wild type (WT), characterized by deeper green leaves and significantly increased flower size, leaf area, and stem diameter (Fig. 1C, Fig. S4). These phenotypic changes are consistent with traits associated with polyploidy in watermelon. Flow cytometry analysis revealed that both *Closd1-1* and *Closd1-2* mutants exhibited doubled ploidy compared to WT plants (Fig. 1E, Fig. S4). Polyploidy can arise from two distinct abnormal cell cycles during mitosis: endoreplication and endomitosis. Endomitosis results in the doubling of the chromosome number, while endoreplication increases the DNA content within each chromosome [5]. Immunofluorescence measurements using an antibody against ClCenH3 were conducted on all *Closd1* mutants to confirm the number of chromosome (Fig. S5). Statistical analysis revealed that the WT plants had an average chromosome count of 20.4 ± 1.1 (*n* = 36), whereas the *Closd1-1* and *Closd1-2* mutants exhibited chromosome counts of 36.1 ± 3.1 (*n* = 35) and 37.5 ± 3.0 (*n* = 39), respectively (Fig. 1I, Fig. S5). The chromosome counts in all *Closd1* mutants were nearly double those of the WT plants, suggesting that the increased ploidy in the mutants is due to endomitosis rather than endoduplication. Notably, tissue culture-induced chromosome doubling is commonly observed in watermelon [7]. To exclude the possibility that the chromosome doubling observed in the mutants was solely due to tissue culture, we established tissue culture-only and empty vector control groups alongside the gene knockout vector transformations. These controls confirmed that the rate of tissue culture-induced doubling was only 7.8–8.9% (Fig. S3, Table S2). Consequently, these findings suggest that the chromosome doubling observed in all eight mutants was directly attributable to mutations in the *ClOSD1* gene, rather than the tissue regeneration process.

These findings indicate that chromosome doubling occurs uniformly across all cells in either homozygous or heterozygous *Closd1* mutants, resulting in tetraploidization rather than the ectopic endomitosis observed in the individual cells of the model plant *A. thaliana Atosd1* mutants. This highlights a unique mechanism for specifically regulating cellular chromosome doubling within watermelon.

Moreover, the disruption of the *OSD1* gene in *Arabidopsis* results in unreduced (100% in males and 85% in females) gametes. To ascertain whether watermelon *Closd1* mutants also generate unreduced microspores, we first conducted Alexander staining on both meiotic immature microspore cells and mature pollen grains from all mutant plants. The findings revealed that while WT plants yielded viable tetrad microspores, male meiosis in *Closd1-1* homozygous mutants resulted in 100% (*n* = 93) dyad formation, as supported by the male meiotic chromosome spreads (Fig. S7). In contrast, *Closd1-2* mutants produced 54.3% (*n* = 96) viable dyads (Fig. 1G and H, Fig. S6), indicating that the heterozygous mutation of *Closd1* partially affects dyad formation. Furthermore, the *Closd1* mutant pollen displayed a notably larger size compared to the WT counterpart (Fig. 1J, Fig. S8). To quantify this observation, we measure the average diameter of pollen grains. The average diameter of wild-type pollen was 59.80 ± 2.64 μm (*n* = 45), whereas the *Closd1-1* mutant pollen had a diameter of 92.20 ± 4.19 μm (*n* = 59). The heterozygous mutant *Closd1-2* exhibited two distinct pollen sizes, with diameters of 93.02 ± 3.19 μm and 73.98 ± 1.15 μm (*n* = 73) (Fig. 1I). Flow cytometry analysis further revealed that the pollen from *Closd1-1* or biallelic mutants was tetraploid, while the pollen from heterozygous *Closd1-2* plants exhibited both diploid and tetraploid ploidy states (Fig. 1K). These results indicate that the mutation of the *ClOSD1* gene in watermelon also leads to the formation of unreduced male gametes.

All self-pollination events in homozygous mutants proved unsuccessful. To better evaluate the ploidy and fertility of female gametes, we conducted reciprocal crosses between WT and *Closd1* mutants. When the *Closd1-1* and *Closd1-6* mutants were used as maternal parents, their fruit development was slower compared to the wild type, and the fruit surface exhibited wrinkling. Only few aborted, empty-shelled seeds were produced (Fig. 1L). All crosses using these two materials as the pollen donor were unsuccessful, indicating that there are issues with the germination of tetraploid pollen. When the *Closd1-2* heterozygous line was used as the female parent, the fruit development was relatively normal, but only 2.4% (5/206) of the seeds produced were plump, while the rest were empty-shelled (Fig. S9). After germination, these five viable seeds were identified as triploids. We speculate that in the heterozygous mutant, female gametogenesis during meiosis produced predominantly tetraploid gametes and a small number of diploid gametes. When these gametes fertilized with haploid wild-type pollen, they resulted in pentaploid and triploid offspring. Since there have been no documented cases of viable pentaploids in watermelon, we infer that the nonviable empty seeds were all pentaploids.

In our study, we found that both homozygous and heterozygous mutations of the *ClOSD1* gene lead to somatic chromosome doubling. Homozygous mutations produce 100% unreduced gametes, while heterozygous mutants only produce a portion of unreduced gametes. We speculate that this phenomenon occurs due to a dosage-dependent effect of the key cell cycle regulator OSD1 in both somatic and reproductive cells. Somatic cells are more sensitive to the levels of OSD1 protein, whereas reproductive cells can partially tolerate a reduction in OSD1 protein levels. We hypothesize that during somatic cell division, cytokinesis occurs after each division. However, in meiosis, the entire process takes place within meiocytes, with cytokinesis only occurring during tetrad formation. Therefore, the syncytial nature of meiocytes ensures that some meiotic divisions in heterozygous mutants can complete meiosis normally.

In conclusion, our study presents the first evidence that mutations in the *ClOSD1* gene in watermelon lead to somatic chromosome doubling, concurrently leading to the production of unreduced gametes. This discovery sheds light on the challenge of generating *MiMe* apomictic lines using the *ClOSD1* gene in watermelon. However, it is noteworthy that other genes, including *AtPS1* and *AtTAM*, have been implicated in unreduced gamete formation in previous studies [4, 8]. Recent reports have demonstrated the successful development of a *MiMe* system in tomato by employing the *SlTAM* gene instead of the *SlOSD1* gene, in conjunction with *SlSPO11-1* and *SlREC8* genes, to produce unreduced clonal gametes [9]. The unavailability of *SlOSD1* in tomato *MiMe* system is attributed to the occurrence of somatic chromosome doubling caused by the mutation of *SlOSD1*, resulting in tetraploid T0 generation plants [9]. Similarly, the tomato *SIUVI4* gene mutant causes an increase in nuclear DNA ploidy [10], which is a phenomenon similar to that observed in watermelon. Therefore, it is crucial to investigate alternative genes to *OSD1* for establishing a *MiMe* system in watermelon and potentially in other Cucurbitaceae species too. Our study lays the principal foundation for regulating watermelon ploidy.

## Supplementary Material

Web_Material_uhae288

## Data Availability

The supplementary data and methods generated in this study are available on the Figshare database under the accession https://figshare.com/s/7d403852af503409bed7. The additional data related to this research can be requested from the authors.
