# Supplementary material for: Disruption of ClOSD1 leads to both somatic and gametic ploidy doubling in watermelon
Source: Hortic Res. 2024 Oct 15;12(1):uhae288. doi: 10.1093/hr/uhae288 (PMC11775614; doi:10.1093/hr/uhae288)
Supplement: Web_Material_uhae288 [file web_material_uhae288.zip › Web_Material_uhae288.pdf]

Supplementary figures, tables, and methods for

Disruption of *ClOSD1* leads to both somatic and gametic ploidy doubling in watermelon

A

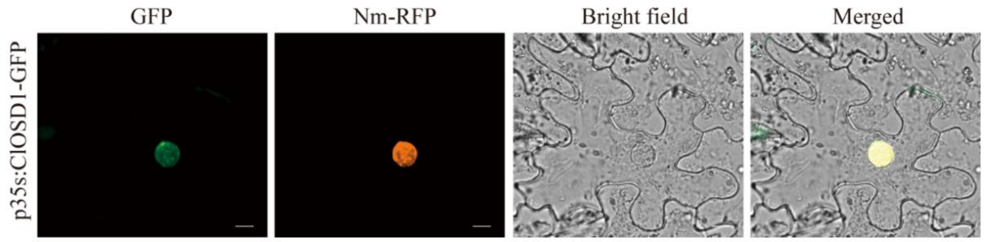

B

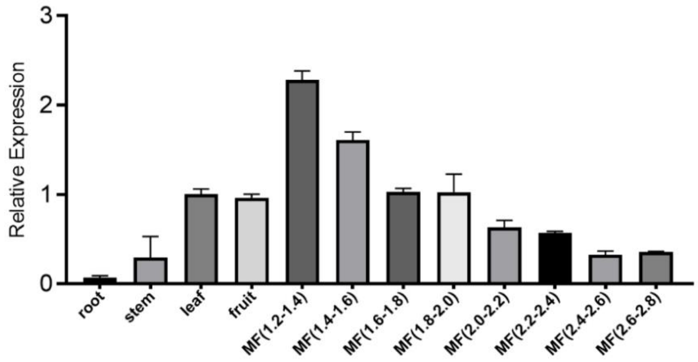

Figure S1: Analysis of *ClOSD1* gene expression patterns in watermelon

A. Subcellular localization of the ClOSD1 protein in tobacco leaf cells. Nm-RFP is used as a nuclear localization marker. Scale bars: 20 μm.

B. Relative expression levels of *ClOSD1* in various watermelon tissues. MF: male flower, with numbers indicating bud diameter (mm).

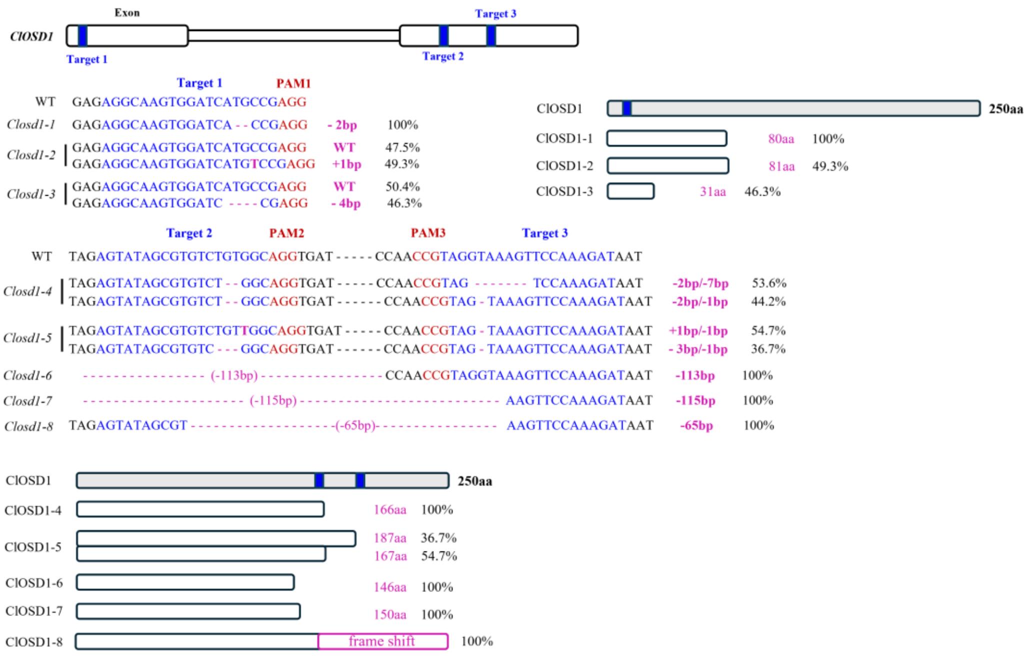

Figure S2: Schematic of the *ClOSD1* gene structure and editing types in *Closd1* mutants

Schematic representation of the *ClOSD1* gene structure, target sequence, CRISPR/Cas9-mediated editing types of the *ClOSD1* gene, and the resulting mutant proteins in watermelon. Genotype analysis was performed using high-throughput sequencing technology.

For Review Only

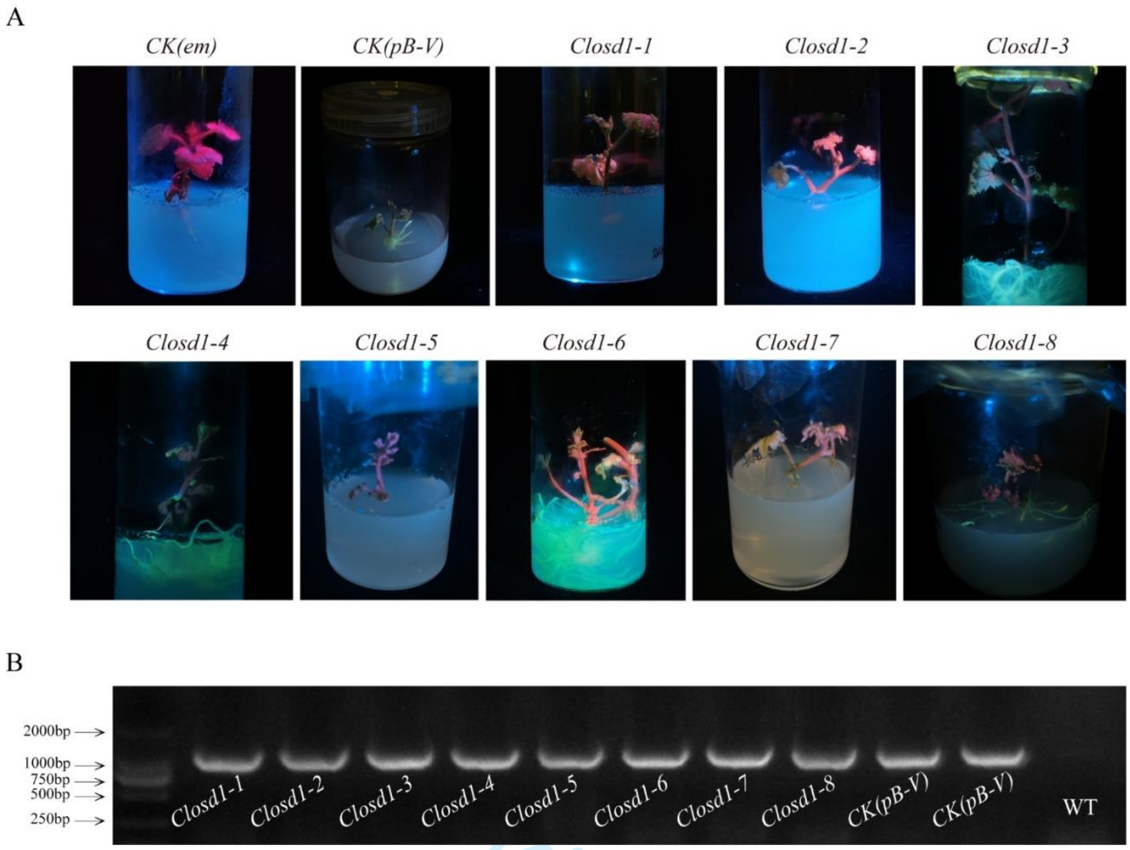

Figure S3: *ClOSD1* gene-editing vector, empty vector transformation, and pure tissue culture-regenerated plant controls

A. Tissue culture seedlings (control and *Closd1* mutants) under excitation light with GFP fluorescent protein. Genotype analysis was conducted using high-throughput sequencing technology. *CK(em)*: Contains no transgenes; *CK(pB-V)*: Contains empty vector without sgRNAs.

B. Detection of T-DNA insertions in *Closd1* mutants and controls.

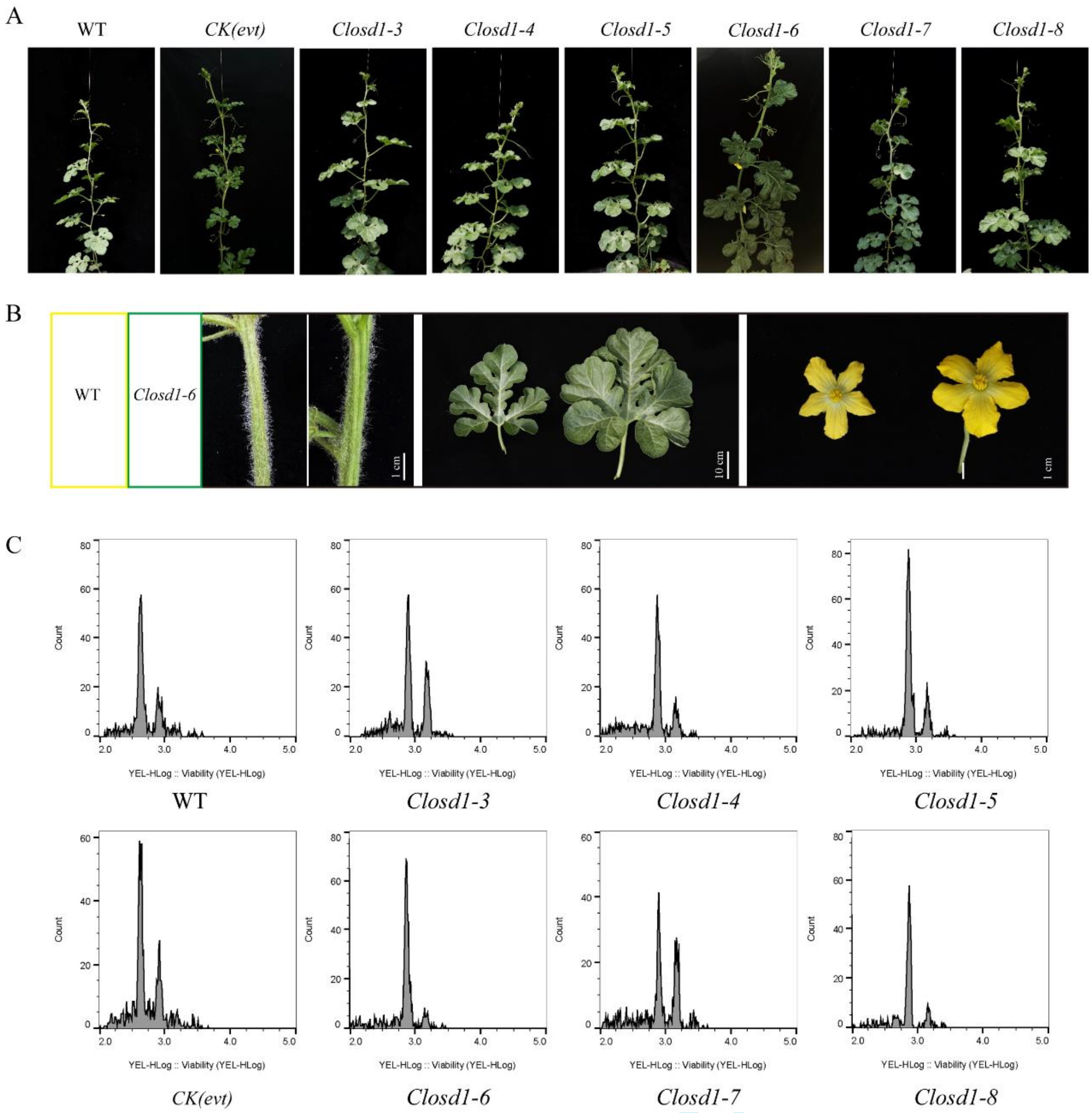

Figure S4: *ClOSD1* mutation leads to somatic doubling in plants

A. Comparison of phenotypes among WT, CK, and *Closd1* mutant plants.

B. The *Closd1-6* mutant was used as a representative plant to compare stems, leaves, and flowers with the WT plants.

C. Flow cytometric analysis of leaves from all *Closd1* mutants and WT plants.

A

ClCENH3 Protein：
MARGRHPAQRKSNRMPSGTGSAQSSPAAPSTPLSGRTQSVGQAQSS
PLRTTKKKKRFRPGTVALREIRNLQKSWNLLIPASCFIRAVKEVSYQL
APQITRWQAEALVALQEAAEDFLVHLFEDTMLCAIHAKRVTIMKKD
FELARRLGGKGRPW

B

C

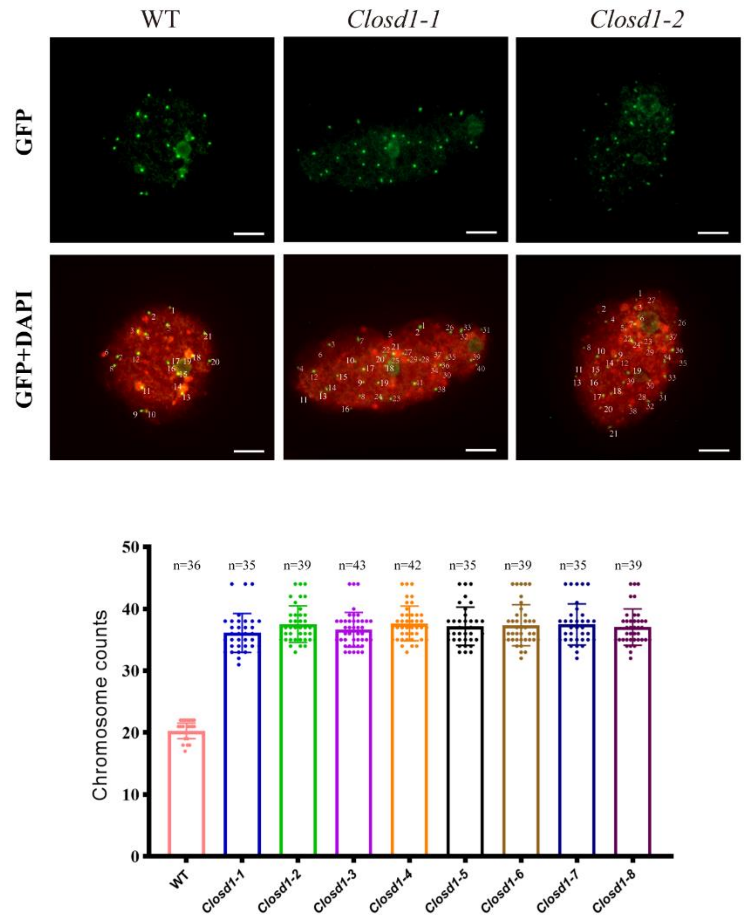

Figure S5: Immunofluorescence hybridization assay using ClCENH3 antibody to determine chromosome numbers in *Closd1* mutants

A. Protein and antibody sequence of ClCenH3 in watermelon. The blue shading indicates the antibody sequence.

B. ClCenH3-mediated immunofluorescence assay in watermelon, showing results for *Closd1-1*, *Closd1-2* mutants (selected as representative cases), and WT plants. The diploid WT had 22 chromosomes (2n=22), while the *Closd1* mutant displayed 44 chromosomes (4n=44). Scale bars: 5 µm.

C. Statistical analysis plots of chromosome counts in different *Closd1* mutants.

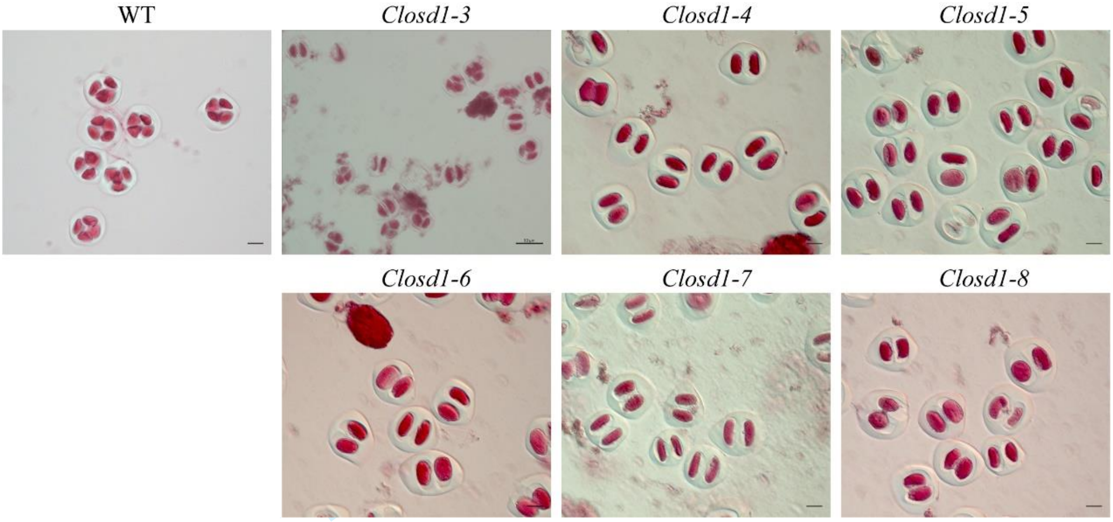

Figure S6: Alexander staining was used to confirm the tetrads produced at the end of meiosis in the wild type and dyads in the *Closd1* mutants. Scale bars: 20 µm.

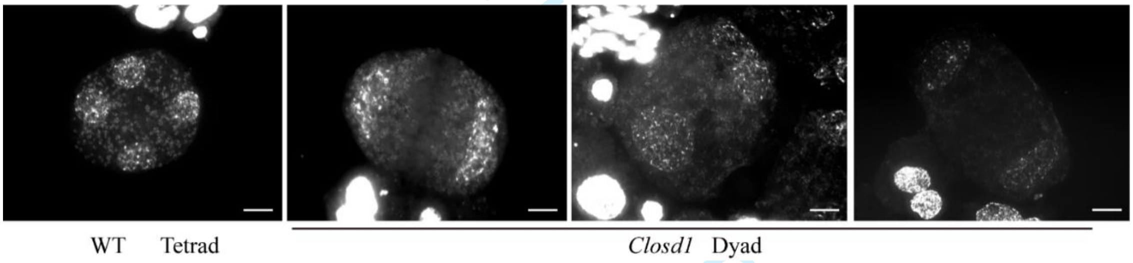

Figure S7: Chromosome spreads of male meiosis telophase II in WT and *Closd1* mutants. Scale bars: 20 µm.

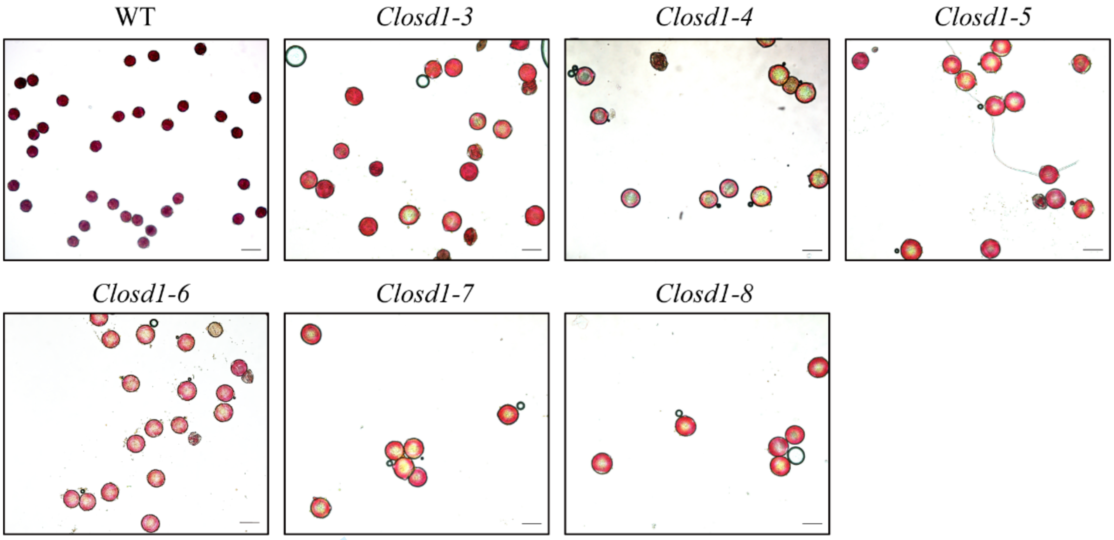

Figure S8: Pollen viability of different *Closd1* mutants was analyzed by Alexander staining. Scale bars: 100 μm.

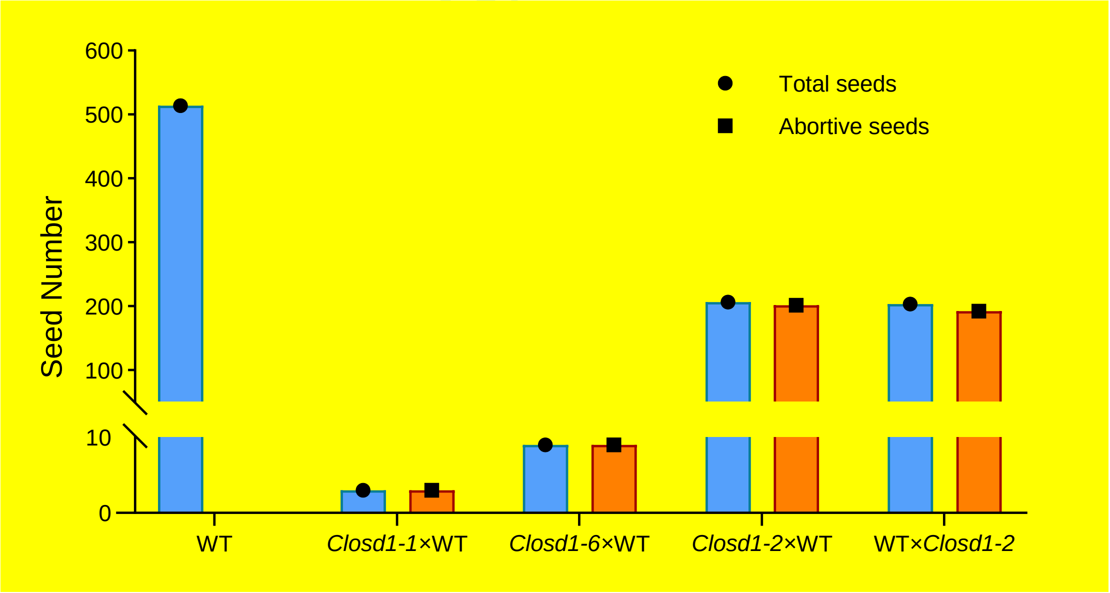

Figure S9: Seed number and abortive phenotype of each hybrid of the *Closd1* mutants.

Supplemental Table 1. Potential off-target sites of three SgRNA of *ClOSD1* gene in watermelon.

Potential off-target site	Sequence	MMs	Locus	Gene ID	Region
ClOSD1-gRNA-1	AGTATAGCGTGTCTGTGGCAGG				
ClOSD1-off target1-1	ACTATAGCTTCTCTGTTGCAGG	4MMs	Chr2:-31053027	Cla013251	non
ClOSD1-off target1-2	ATTACAATGTGTCTGTGGCAGA	4MMs	Chr2:+20212058	Cla013340	cds
ClOSD1-gRNA-2	ATCTTTGGAACTTTACCTACGG				
ClOSD1-off target2-1	ATCTCTATAACTTTACCTATGG	4MMs	Chr11:+25567045	Cla016871	non
ClOSD1-off target2-2	TTCCTTGGAACTTAACCTATGG	4MMs	Chr5:+30300101	Cla020384	non
ClOSD1-off target2-3	AACTTTGGAAATTTACCAAAAG	4MMs	Chr3:+9174996	Cla019732	cds
ClOSD1-gRNA-3	AGGCAAGTGGATCATGCCGAGG				
ClOSD1-off target3-1	AAGCAAGAGGGTCATACCGTGG	4MMs	Chr11:-15259317		Intergenic
ClOSD1-off target3-2	TATCAAGTGGATCAAGCCGAGG	4MMs	Chr4:+6246412	Cla011643	non
ClOSD1-off target3-3	ATACGAGTGGATCATGCCAAGG	4MMs	Chr5:16233401		Intergenic
ClOSD1-off target3-4	AAGCAAGAGGATCATGTCTAGG	4MMs	Chr2:-37491965		Intergenic

Supplemental Table 2. Comparison of the tetraploidization rates of regenerated shoots during tissue culture, empty vector, and *ClOSD1* editing vector transformations in watermelon.

Transformation event	Transformation vecter	Total number (adventitious buds + plants)	Tetraploid	Tetraploid rate (%)
CK(nt)	-	89	7	7.8 %
CK(pB-V)	pBSE402	56	5	8.9 %
Closd1	pBSE402-SgRNA	21	21	100 %

Note: In addition, all regenerated positive shoots that did not undergo editing of the *ClOSD1* gene in pBSE402-SgRNA vector transformation were not tetraploid.

For Review Only

Supplemental Table 3. Primers used in this study.

Experiment	Primer	Sequence(5'-3')
For qPCR	qPCR*Actin*-1F	CCTACAACTCAATTATGAAGTGTG
	qPCR*Actin*-1R	GAAATCCACATCTGCTGGAAGGTG
	qPCR*Actin*-2F	GTCGTACAACAGGTATTGTG
	qPCR*Actin*-2R	AAGGTCCAGACGGAGGATAG
	RT*ClGIG1*-1F	GATCCGAAGGTATATTGGACGAG
	RT*ClGIG1*-1R	GGGATTCGTAGGTCTTTGTTGAG
	RT*ClGIG1*-2F	GCCAAGGAAGTGACAATAGTC
	RT*ClGIG1*-2R	TCTTCTGGGGAGTGAGAGTCT
For RNA in situ	*ClGIG1*-T7F	TGTAATACGACTCACTATAGGGCCAAGAGATAGATTGGAGAGGCA
hybridization	*ClGIG1*-SP6R	GATTTAGGTGACACTATAGAATGCTGAGAGGGGTTCTTGGGTACCAA
For subcellular	*ClGIG1*-pGreen-F	TTCCTGCAGCCCGGGATGTCGGAAGCAAGAGATAGATTGG
localization	*ClGIG1*-pGreen-R	TTCTCCTTTACTCATACTAGTGGATCCACGAAACGACATTA
	Ins-Pg-F	CATTTGGAGAGGACAGCCCA
	Ins-Pg-F	AGAATTGGGACAACTCCAGTG
For gene editing	*ClGIG1*-gRNA1-F	TCGAAGTAGTGATTGAGTATAGCGTGTCTGTGGCGTTTTAGAGCTAGAAATAGC
And testing	*ClGIG1*-gRNA1-R	TTCTAGCTCTAAAACTAGGTAAAGTTCCAAAGATCAATCTCTTAGTCGACTCTAC
	ClGIG1-SgRNA3-F	ATTGAGGCAAGTGGATCATGCCG
	ClGIG1-SgRNA3-R	AAACCGGCATGATCCACTTGCCT
	U626-IDF	TGTCCCAGGATTAGAATGATTAGGC
	U629-IDR	AGCCCTCTTCTTTCGATCCATCAAC
	gRNA1JC-F	TCAATATCACCTGTGTTTTCCA
	gRNA1JC-R	TCAACGAAACGACATTAAAGT
	gRNA3JC-F	ATGTCGGAAGCAAGAGATAGA
	gRNA3JC-R	CGTATATGCATACCAAAGACC
	G1-HITOM-F	GGAGTGAGTACGGTGTGCAGAGAAAACGAAGGCCAAGGA
	G1-HITOM-R	GAGTTGGATGCTGGATGGGGAGTTCAAGAGCTTCTTCT
	G3-HITOM-F	GGAGTGAGTACGGTGTGCATGTCGGAAGCAAGAGATA
	G3-HITOM-R	GAGTTGGATGCTGGATGGATTCGTAGGTCTTTGTTGAGC
For T-dna insertion	Cas9-IDF	TCCGAGTTCTCCAAGCGCGTGA
testing	Cas9-IDF	TCGAGCCATCCGGAAGATGAATTC
	U6P-IDF	CTTCAAAAGGCCCCTGGGAATCTGA
	U6T-IDR	AACCCCAGAAATTGAACGCCGAAG
For sgRNA	OffT1-1F	TTCCAGGGAGTATGAGCAAGG
off-target detection	OffT1-1R	TGATGGTCCATCTGACTCCAT
	OffT1-2F	TATCGTGGTCTATTGCAGACAG
	OffT1-2R	CTGTCCACGTTCTCTCTCTTT
	OffT2-1F	TAGGGGATGGATTCCATAAGC
	OffT2-1R	TTGGATCACAGAATCCAGACG
	OffT2-2F	GACGTAGTTCAGTTGCTGCAG
	OffT2-2R	GACGTACCATTGGACCAACAG
	OffT2-3F	ACTTGACTTAGCTCCACCGTA

For Review Only

OffT2-3R	AGGAAGTGGATTGTCTGAAG
OffT3-1F	CATGATCACACTTGAGTAATTCT
OffT3-1R	CAAGGAGTGTACCTGTCACCAC
OffT3-2F	GACGTAGATGATGAATAGGCACA
OffT3-2R	ATATGCACCATGACATTGGTCATG
OffT3-3F	TTGTCCATTGAATAACCATAT
OffT3-3R	AGATCGAGTCGCACATGATCAA
OffT3-4F	ATGCTGACCATAGAGAACAA
OffT3-4R	CAGTTGTCTTGCAGATGGACAAC

For Review Only

Materials and methods

Plant material

The wild-type watermelon materials used in this experiment were “YL” and “TC”, which were obtained and stored in our research group. All the mutants and wild type materials were planted in the experimental field of Northwest A&F University, Yangling.

In situ hybridization of male anther development stages

Flower buds fixation, chromosome spreads, and in situ hybridization were performed as previously described[1,2] with minor modifications. A 353bp RNA probe was designed based on the cDNA sequence of *ClGIG1*. And DIG RNA Labeling Kit (Roche, Rotkreuz, Switzerland) was used to label the sense and antisense probes of *ClGIG1*, according to the manufacturer's instructions. Male flower buds were vacuumed in 4% paraformaldehyde and fixed at 4℃ for 24 hours. After dehydration, xylene wax was soaked at 42℃, and then the sample was embedded and sliced after the material was cooled naturally. Note that the slide used at this time must be guaranteed to be RNase free. Samples were incubated in prehybridization buffer: 6 x SSC, 0.1% SDS, 50% formamide, 100 μg/mL tRNA at 42℃ for 3 hours and DIG-labeled RNA probes were added at 42℃ for overnight hybridization. Incubate in BCIP/NBT solution and finally observe the signal.

Transformation of watermelon

The watermelon explants were transformed following a previously described[3] method with slight modification. Briefly, seeds were sterilized with 3% sodium hypochlorite and 75% alcohol, then placed on a basic MS solid culture medium for seeding for 3 days, and the watermelon cotyledon was cut into about 1.5 × 1.5-mm small pieces. For transformation using *Agrobacterium tumefaciens* strain EHA105, watermelon cotyledons were immersed in diluted Agrobacterium solution (OD600=0.6-0.8) for 15 minutes. Cotyledon explants were co-cultured in the dark for 4 days and then transferred to selective induction medium containing 1.5mg/L 6-BA, 200mg/L Timentin. And maintained under standard photoperiod conditions (28°C, 16 hours daylight, and 8 hours dark) for 2 weeks. The medium was refreshed every 10 days. Adventitious buds were excised and transferred to bud elongation medium containing 0.1mg/L 6-BA, 0.01mg/L NAA, and 200mg/L Timentin. Finally, the buds were transferred to rooting medium. The success of the transformation was evaluated using a handheld lamp to screen the plants.

Genotyping of transgenic watermelon lines

Extracted genomic DNA from each T_0 transgenic leaf tissue. PCR amplification of the target genomic region was performed using specific primers, and the PCR products were sequenced using Sanger sequencing. Additionally, select a subset of PCR products with high-quality

For Review Only

sequencing data and perform deep sequencing on the Hi-Tom(High-throughput mutation) platform. The Hi-Tom strategy simplifies the construction of diverse pooled sequencing libraries by requiring only two common PCR steps[4]. Design primers with adapter sequences to amplify the target fragment. Comprehensively analyze the sequencing data by uploading it to the Hi-TOM online analysis website (Hi-TOM: http://www.hi-tom.net/hi-tom/index-CH.php), to determine the specific editing type of each plant at each site.

Off-target analysis

Utilize the CRISPR-P v2.0 (http://crispr.hzau.edu.cn/cgi-bin/CRISPR2/CRISPR) website for designing target sites and predicting potential off-target sites. Subsequently, identify potential off-target sites on the Cucurbit Genomics Database (http://cucurbitgenomics.org/v2/) using specific primers (Table. S3), amplify them, and sequence them to assess the off-target potential.

Immunofluorescence assay

Based on the CenH3 protein sequence of *Arabidopsis thaliana*, blast ClCenH3 protein sequences against the Cucurbit Genomics Database. ClCenH3 protein antibody was raised against the N terminus protein sequence, which was manufactured by Genscript (Hangzhou)(Fig. S5). We performed this procedure as previously reported[5] with minor modifications. The cell suspension and sucrose solution were dropped 1:1 on a slide and dried overnight at room temperature. Washed twice with 1 x PBS buffer, added 100μL 1st antibody solution (2% BSA in 1 x PBS with 0.1% Tritonex-100:1st antibody = 200:1). The slides were incubated overnight in a wet box at 4℃. Washed twice with 1 x PBS buffer. 100μL of 2nd antibody solution was dropped onto the slide, then left at high humidity at 37℃ for 1 hour. Dehydrated with 70%, 90% alcohol and dried the slides at room temperature. Samples were stained with DAPI.

Flow cytometric assay

Samples were prepared as described[6,7] with minor changes. Particularly, fresh leaves or pollen were placed in cell lysate LB01 buffer. The leaves and pollens were continuously chopped vertically with a sharp blade to shatter the cells. Nuclear solution was collected through a 35 μm cell filter, stained with 50 μg/mL PI (Propidium Iodide), and analyzed on Muse Cell Analyzer (Luminex). Typically, 2000 nuclei were analyzed per sample. The DNA content of the mutant was calculated according to the location of 2C or 4C peaks of the wild type and mutant.

Cytological Analysis of Male Meiosis

Flower buds of different sizes were taken according to the male flower reproductive calendar of watermelon, fixed in Carnoy's Fluid, and stored at -20℃ as possible. we performed this procedure as previously reported[8,9]. The flower was peeled and washed three times in 0.01M

For Review Only

citrate buffer (4 mL of 0.1 M citric acid, 6 mL of 0.1 M sodium citrate, pH 4.5) for 5 min then treated with 2% cellulase (Sigma-Aldrich) and 0.25% pectinase (Sigma-Aldrich) in citrate buffer for 3 hour in a moist chamber at 37°C. The anthers were crushed with tweezers, each slide was added with 10μL 60% acetic acid solution, covered with the cover slide, pressed vertically with thumb, frozen at -80°C for 10min, removed the cover slide, dried at room temperature, and stained with DAPI.

References

1. Li, X.R. *et al.* Arabidopsis DAYU/ABERRANT PEROXISOME MORPHOLOGY9 is a key regulator of peroxisome biogenesis and plays critical roles during pollen maturation and germination in planta. *Plant Cell* (2014) **26**, 619-35.
2. Moran, E.S., Armstrong, S.J., Santos, J.L., Franklin, F.C.H. & Jones, G.H. Chiasma formation in Arabidopsis thaliana accession Wassileskija and in two meiotic mutants. *Chromosome Res* (2001) **9**, 121-128.
3. Tian, S. *et al.* Efficient CRISPR/Cas9-based gene knockout in watermelon. *Plant Cell Rep* (2016) **36**, 399-406.
4. Sun, T., Liu, Q., Chen, X., Hu, F. & Wang, K. Hi-TOM 2.0: an improved platform for high-throughput mutation detection. *Sci. China Life Sci* (2024) **67**, 1532-1534.
5. Liu, Q. *et al.* AtRKD5 inhibits the parthenogenic potential mediated by AtBBM. *J. Integr. Plant Biol* (2024) 66, 1517–1531.
6. Kron, P. & Husband, B.C. Using flow cytometry to estimate pollen DNA content: improved methodology and applications. *Annals of Botany* (2012) **110**, 1067-1078.
7. Borges, F. *et al.* FACS-based purification of Arabidopsis microspores, sperm cells and vegetative nuclei. *Plant Methods* (2012) **8**, 44.
8. Ross, K.J., Fransz, P. & Jones, G.H. A light microscopic atlas of meiosis in Arabidopsis thaliana. *Chromosome Res* (1996) **4**, 507-16.
9. Tian, S. *et al.* A 2.09 Mb fragment translocation on chromosome 6 causes abnormalities during meiosis and leads to less seed watermelon. *Hortic Res* (2021) **8**, 256.

For Review Only
